# Nutritional imbalance suppresses migratory phenotypes of the Mongolian locust (*Oedaleus asiaticus*)

**DOI:** 10.1098/rsos.161039

**Published:** 2017-06-07

**Authors:** Arianne J. Cease, Jon F. Harrison, Shuguang Hao, Danielle C. Niren, Guangming Zhang, Le Kang, James J. Elser

**Affiliations:** 1 School of Sustainability, Arizona State University, Tempe, AZ 85287, USA; 2 School of Life Sciences, Arizona State University, Tempe, AZ 85287, USA; 3 State Key Laboratory of Integrated Pest Management, Institute of Zoology, Chinese Academy of Sciences, Beijing 100101, People Republic of China; 4 State Key Laboratory of Vegetation and Environmental Change, Institute of Botany, Chinese Academy of Sciences, Beijing 100093, People Republic of China

**Keywords:** migratory polyphenism, plant–insect interactions, nutrition, grasslands, migration, movement ecology

## Abstract

For many species, migration evolves to allow organisms to access better resources. However, the proximate factors that trigger these developmental changes, and how and why these vary across species, remain poorly understood. One prominent hypothesis is that poor-quality food promotes development of migratory phenotypes and this has been clearly shown for some polyphenic insects. In other animals, particularly long-distance bird migrants, it is clear that high-quality food is required to prepare animals for a successful migration. We tested the effect of diet quality on the flight behaviour and morphology of the Mongolian locust, *Oedaleus asiaticus*. Locusts reared at high population density and fed low-N grass (performance-enhancing for this species) had enhanced migratory morphology relative to locusts fed high-N grass. Furthermore, locusts fed synthetic diets with an optimal 1 : 2 protein : carbohydrate ratio flew for longer times than locusts fed diets with lower or higher protein : carbohydrate ratios. In contrast to the hypothesis that performance-degrading food should enhance migration, our results support the more nuanced hypothesis that high-quality diets promote development of migratory characteristics when migration is physiologically challenging.

## 1. Introduction

Migration is an impressive and prevalent component of the life history of many organisms [1]. Birds, fish, mammals and insects in groups of up to millions of individuals move over vast areas of the globe, connecting distant countries and transferring immense quantities of nutrients [2]. These large-scale movements have implications for conservation [3]. If the migrating individuals are pests, such as locusts, food security may be threatened [1,4]. Migration is often viewed as an adaptation to shifting or patchy environments, with migration being induced to allow individuals to seek out better habitats at a given time and life stage [1,5]. However, why and how migration evolves across taxa and the environmental factors that induce migration remain opaque [5,6].

One prominent hypothesis is that poor-quality food promotes the development of migratory phenotypes [7,8], and this has been clearly shown for some polyphenic insect species. For example, Denno *et al*. showed that there was an interactive effect between plant nutrient content and population density in the wing dimorphic planthopper *Prokelisia marginata*, which migrate fairly short distances of 3 km or less [9]. Feeding on plants that had been heavily fertilized (nitrogen, phosphorus and potassium) increased adult size and fecundity and also suppressed the development of long-winged, migratory morphs at high density. High nitrogen (or other nutrient) content is often equated implicitly with ‘high quality’. However, increasing evidence suggests the relationship between dietary nutrients and consumer performance is typically nonlinear (reviewed in [10]). Therefore, for this paper we define high quality as performance-enhancing for the consumer (e.g. increased fitness or fitness correlates such as growth rate, survival or reproduction). Aphids are a classic example where poor food quality has been implicated in promoting development of long-winged adults and migration [11]. This pattern has been shown for some but not all aphid species. Müller *et al.* [12] reviewed 38 studies on aphids and found that 34% of these supported the hypothesis that performance-degrading diets increase production of winged morphs. There are also vertebrate examples. Female mule deer (*Odocoileus hemionus*) with lower body fat were more likely to migrate earlier to a lower Sierra Nevada winter range area (20–40 km), possibly to decrease risk of thermoregulatory and locomotive costs if caught in substantial snow [13].

In other animals, particularly long-distance migrants, it is clear that high-quality food is required to prepare animals for a successful migration. Long-distance migration is expensive and requires, at least in some taxa, increases in muscle size and fuel stores (reviewed in [1]). Multiple studies examining bird migration have concluded that high-quality food is an important determinant of a migrant's flight capacity [14–18]. Many migrating birds and insects forage while migrating and take advantage of winds to reduce energy expenditure during flight [14,16,19–21]. Some insect studies have found that high-quality food promoted migratory behaviour. Sappington & Showers [22] found that low-quality larval or adult diets reduced flight propensity and migratory duration in the moth *Agrotis ipsilon* [22]. This moth has been recorded to fly roughly 1000 km within a few days; however, the flights are assisted at least partially by wind currents [23,24]. Similarly, the strawberry aphid (*Chaetosiphon fragaefolii*) were more likely to produce winged offspring when fed performance-enhancing host plants [25]. Plausibly the effect of food quality on migration may depend on the physiological challenge of the migration; with many birds exemplifying the need for high-quality food for successful migration and wind-blown small insects being paradigms of low-cost migrations in which flight allows escape from poor local conditions.

We used a series of laboratory- and field-based studies, and plant and artificial diets, to test the commonly held prediction formalized by Johnson [7,8] that performance-degrading food, in conjunction with high local population density, should enhance migratory characteristics. We studied the Mongolian locust, *Oedaleus asiaticus* (Orthoptera: Acrididae, *O. decorus asiaticus* Bei-Bienko, 1941), which flies for more than 2 h and 15 km in laboratory conditions [26] and likely more than 100 km within a few days' time in the field [27]. This non-model locust [28] provides an excellent model for testing interactions between food quality and population density. Outbreaks and migratory swarms are composed mostly of brown morphs that have increased relative investment in their thorax and hind legs when compared with the green, non-outbreak morphs [29]. Tests with N-fertilized plants and artificial diets have previously shown that brown *O. asiaticus* prefer, grow and survive best on low-N plants and artificial diets with a 1 : 2 mass ratio of protein : carbohydrate [30]. Thus, low-N plants are performance-enhancing for *O. asiaticus*. What remains unknown is whether the performance-enhancing plants induce or suppress development of the migratory phenotype. To answer this, we confined locusts to low or high density in either laboratory or field cages and fed them host plants from control or N-fertilized treatment plots. We then measured changes in growth performance and morphology, including colour, in the locusts. We complemented the experiments using host plants with a synthetic diet experiment to directly manipulate macronutrient balance and then measure flight activity. In sum, these experiments allowed us to address the question of how plant quality interacts with local density to influence migratory polyphenism and flight activity in this economically important locust.

## 2. Material and methods

### 2.1. Field site and animals

Field experiments were conducted near the Inner Mongolia Grassland Ecosystem Research Station in the Xilin River Basin, Inner Mongolia Autonomous region, China (43°38′ N, 116°42′ E, 1100 m.a.s.l.). These studies were carried out in June–August of 2008 (laboratory rearing experiments), 2009 (field cage rearing experiments) and 2013 (synthetic diet and flight experiments). *Oedaleus asiaticus* (*O. decorus asiaticus* Bei-Bienko, 1941) were collected from a grazed pasture near the research station. This species hatches in early June, undergoes five juvenile stages, and then moults into adults in mid-July [31]. Mitochondrial genome analyses have shown that *Oedaleus asiaticus* is closely related to the migratory locust, *Locusta migratoria* (Linnaeus 1758), which has migratory swarms throughout Asia, Africa and Australia [32]. We designed the timing of our experiments to coincide with when we could find *Oedaleus asiaticus* nymphs in the field. Inner Mongolia is representative of much of the Eurasian Steppe region with grasslands dominated by *Stipa grandis* and *Leymus chinensis* [33].

### 2.2. Effects of N-fertilization and density on laboratory-reared locusts

We collected fourth-instar brown female locust nymphs from high-density field populations (50 + nymphs m^2^) and randomly assigned them to control or N-fertilized food plant treatment groups and either low (1 locust cage^−1^) or high (8 locusts cage^−1^) density on day one of the fifth and final larval instar. Cages were 10 × 10 × 15 cm and made of 1 mm^2^ cloth mesh (smaller than the field cages, and therefore the densities of animals were more similar in laboratory and field than the number per cage). The locusts were able to see and smell locusts in other cages, as occurred within the field cages. Locusts were kept in an incubator that was set to regulate the following: 14 L : 10 D cycle, 27°C : 25°C, 50% : 40% relative humidity. However, due to frequent power outages, temperature sometimes fluctuated down as much as 15% about every 3 days; however, all treatment groups were exposed to the same temperature fluctuations. Fresh *Leymus chinensis* grass was cut from the either control or fertilized field plots every other day, secured with cotton in glass cylinders containing water, and presented ad libitum, similar to [29]*.* In the fertilized plots, ammonium nitrate (NH_4_NO_3_; 175 kg N ha^−1^ yr^−1^) was applied once in the late spring, just before a heavy rainfall and at least two weeks before plants were collected for this experiment. Control grass (unfertilized) was collected from plots adjacent to the N-fertilized plots. The rate of N fertilization was similar to that used for most crops [34]. Our prior study showed that this N fertilization treatment increased grass protein content for two common grass species at the site (*Leymus chinensis* and *Stipa grandis*) by 4–7%, approximately doubled the N : C ratio of the leaves, and strongly reduced the feeding preference of field-collected *O. asiaticus* for the plants [30].

Specific growth rates (*μ*) were calculated as *μ* = [ln(*M*_2_*/M*_1_)]/d*t*, where *M*_2_ and *M*_1_ are the locust body masses at day 1 after moult to adult and at day 1 of the final juvenile instar, respectively, and d*t* is the number of days spent in the final juvenile instar. For the assessment of adult morphology, within 4 h after moult to adult, we isolated treatment locusts in a cage with no food for 24 h to allow the cuticle to harden. Individuals were weighed to the nearest 0.1 mg, frozen at −20°C, and then dried at 50°C for 3 days. We dissected the locusts after they had dried by removing their gut and separating the head, wings, legs, thorax and abdomen. Body components were weighed to the nearest 0.001 mg using a microbalance. We then relaxed the hind wings using a weak vinegar solution, spread them flat for drying, digitally scanned the wings and then measured wing area using Image J software (resolution = 79 pixels cm^−1^) [35]. All treatment groups contained 10 locusts, but individuals were removed from analyses if their wings were torn or disfigured such that we could not obtain accurate morphological measurements. We only tracked one individual per cage (even for the high-density cages). Closely tracking one individual per cage allowed us to measure growth rate without concerns of pseudoreplication associated with including multiple individuals from the same cage in the analysis.

To compare relative allocation to different body components, we first standardized the variables using a Z-score transformation so that all variables would be weighted evenly in our analysis [36]. Masses of most body components were correlated, so we combined these variables into one linear variable using a maximum-likelihood factor analysis (table 1). Increased values of this migratory morphology index (MMI) represent increased allocation of mass to thorax, wings, hind legs, and, to a lesser extent, abdomen, relative to head.

**Table 1.** Factor loadings for mass allocation to different body components using a maximum-likelihood extraction.

variable	factor loadings
head mass (Z)	0.13
hind legs mass (Z)	0.82
abdomen mass (Z)	0.63
thorax mass (Z)	0.84
wings mass (Z)	0.98
eigenvalue	2.7
% of total variance	55

### 2.3. Effect of N-fertilization and density on locusts reared in the field

We constructed 1 m^3^ cages with iron rod frames and fine cloth mesh to enclose plant communities that were either in control or N-fertilized plots. Spiders and arthropods were removed by hand from the cages prior to adding locusts. Green locusts (with colour defined as in [29]) were collected as third instars from high-density field populations (50 + nymphs m^2^) and randomly assigned to control or N-fertilized plots and either low (20 locusts cage^−1^) or high (100 locusts cage^−1^) density. We selected the high-density treatment levels to be in the range of what has been reported in field observations of migratory swarms of this species (S. Hao 2009, personal communication). Our low-density field treatment was at approximately the density at which we observed primarily green morphs; our observations indicated that the low-density locusts separated themselves within the complex vegetation and had minimal interactions [29]. We selected green morphs because they tend to be non-migratory even when found in higher population densities [29], and we wanted to test the propensity of diet and density to promote locusts switching from non-migratory to migratory phenotypes.

In the fertilized plots, ammonium nitrate (NH_4_NO_3_; 175 kg N ha^−1^ yr^−1^) was applied once in the late spring, just before a heavy rainfall and at least two weeks before the start of this experiment, similar to the laboratory-based experiment. Eight N-fertilized and eight control plots were randomly assigned within one field that had been fenced for 5 years to prevent livestock grazing. There were 1 m paths separating plots and each plot contained four to nine 1 m^3^ locust cages each. We ended the field trial when most locusts were either in the fifth instar or had just moulted to adults and recorded survival and colour on that day.

### 2.4. Effects of varying macronutrient ratios in artificial diets on flight behaviour and survival

We collected green and brown female locusts from high-density field populations during the early fifth instar and transferred them to small (16 × 9 × 11 cm) plastic cages with 17 locusts per cage and one cage per treatment (*N* = 68; high-density rearing conditions). Cages either had all green or all brown morphs. Locust groups were fed one of three synthetic diets (7 : 35, 14 : 28 or 35 : 7 protein %: carbohydrate %), made as described in [30]. Tests with artificial diets have previously shown that *O. asiaticus* prefer, grow and survive best on diets with a 1 : 2 mass ratio of protein : carbohydrate [30]. Therefore, in this experiment we tested whether diets with a 1 : 2 protein : carbohydrate ratio are better at promoting flight behaviour relative to those with lower or higher protein : carbohydrate ratios.

In a previous paper, we were unable to convince *O. asiaticus* to fly [29]. While it is unclear why this locust would not fly on our flight mill, it is possible that the lack of a steady wind stream precluded sustained flight. For the current study, we used a different approach whereby locusts were held in place and a consistent air stream faced directly at their heads was supplied by a fan. Locusts were individually marked when they moulted to adults and flight was tested 4–6 days later. Locusts that did not survive or were not viable for flight (e.g. disfigured wings) were excluded from the analyses. Locusts were suspended in the air using a pin secured to their pronotum with dental gum and induced to fly with a fan that delivered a wind current of 11–13 km h^−1^, a similar wind speed to McAnelly *et al*. [37] and Kent *et al*. [38]. Floodlights were used to raise the air temperature to 30–32°C. We recorded the duration of flight during a 10 min minimum trial. All flight tests were conducted between 15.00 and 21.00 local time because *O. asiaticus* frequently migrates at this time of day [27]. We simultaneously tested one locust from each treatment group to ensure that animals from the various treatment groups experienced similar conditions. We initiated take-off by breaking locust tarsal contact with its roost; this was repeated five times or until the locust flew continuously. To be scored as ‘flying’ both fore and hind wings had to be flapping and we added together the total flight time for all take-off attempts even if locusts stopped in between flight bouts.

### 2.5. Statistics

All data were tested for assumptions of normality and homoscedasticity implicit in parametric tests. All proportional data were arcsine-transformed prior to analysis, and remaining datasets were arcsine- or log-transformed and outliers removed as necessary to meet assumptions for parametric tests. Analyses were performed using Statistica 10 (2011) and SPSS v. 23.

## 3. Results

### 3.1. Effect of N-fertilization and density on laboratory-reared locusts

**Figure 1.**
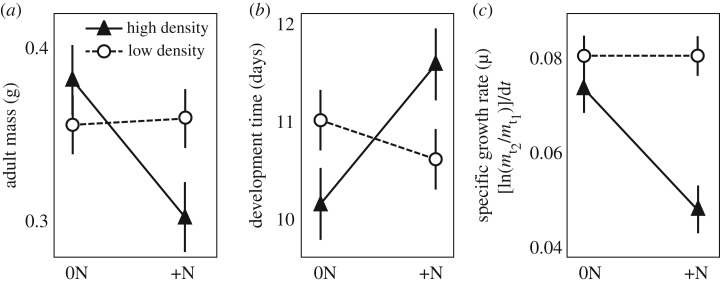
Adult mass (*a*), development time (*b*) and specific growth rate (*c*) in locusts from the laboratory rearing study. Locusts reared at high density and fed low-N plants had the heaviest adult mass, shortest development time and among the highest specific growth rates. There were no differences between locusts reared at low density and fed low-N or high-N plants. Here and throughout all figures, closed triangles indicate high and open circles indicate low locust density; values indicate mean ± s.e.

In general, consumption of unfertilized plants promoted growth and development for high-density locusts. Consumption of N-fertilized leaves reduced performance indices for *O. asiaticus* reared at high density (figure 1). There were significant interactive effects of locust density and host plant N enrichment on adult mass (two-factor ANOVA *F*_1,30_ = 5.24, *p* = 0.03), development time (two-factor ANOVA *F*_1,30_ = 7.66, *p* = 0.01) and mass specific growth rate (two-factor ANOVA *F*_1,30_ = 8.27, *p* = 0.007). Simple main effects analyses showed that, when fed N-fertilized plants, locusts reared at high density, when compared with locusts reared at low density, attained a smaller adult mass (ANOVA *F*_1,15_ = 4.86, *p* = 0.044) and had lower specific growth rates (ANOVA *F*_1,15_ = 31.81, *p* < 0.001) but the extension in development time was not statistically significant (ANOVA *F*_1,15_ = 2.90, *p* = 0.11). When fed unfertilized host plants, locusts reared at high density had a shorter development time than those raised at low density (ANOVA *F*_1,15_ = 6.62, *p* = 0.02), but there were no differences in adult mass (ANOVA *F*_1,15_ = 1.04, *p* = 0.32) or specific growth rate (ANOVA *F*_1,15_ = 1.05, *p* = 0.32). No mortality occurred for individuals observed in this study.

**Figure 2.**
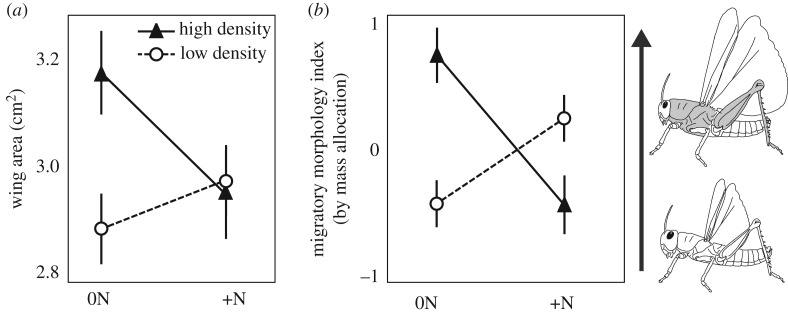
Locusts reared at high density and fed low-N plants had greater relative wing areas (analysed using an ANCOVA with body mass as a covariate) than the three other groups (*a*). Locusts reared at high density and fed low-N host plants had the highest migratory index (*b*). We calculated this index by combining the mass variables for five body components into one linear variable using a maximum-likelihood factor analysis. Higher values in panel (*b*) represent increased mass allocation to the thorax, wings and hind legs, as represented by shaded portions in the locust illustrations. The features in the locust drawings are for illustration purposes only and are not drawn to scale.

The MMI (see Methods for description) was maximized for animals reared at high densities and consuming unfertilized plants. For example, consumption of N-fertilized plants reduced wing area and the MMI at high locust density (figure 2). All morphological variables covaried with mass, so we included mass as a covariate for these statistical analyses. Locust density and host plant N enrichment had an interactive effect on wing area (two-factor ANCOVA *F*_1,29_ = 4.97, *p* = 0.03). Simple main effects analyses showed that, when fed unfertilized host plants, locusts reared at high density had a greater wing area than those reared at low density (ANCOVA *F*_1,14_ = 7.85, *p* = 0.01), but there was no difference between high- and low-density groups when fed N-fertilized plants (ANCOVA *F*_1,14_ = 0.10). There was a significant interactive effect of locust density and host plant N enrichment on MMI (two-factor ANCOVA *F*_1,29_ = 20.56, *p* < 0.001). When fed unfertilized host plants, locusts reared at high density had a higher MMI than those reared at low density (ANCOVA *F*_1,14_ = 16.33, *p* = 0.001), but there was no difference between high- and low-density groups when fed N-fertilized plants (ANCOVA *F*_1,14_ = 3.03, *p* = 0.08).

### 3.2. Effect of N-fertilization and density on field-reared green locusts

**Figure 3.**
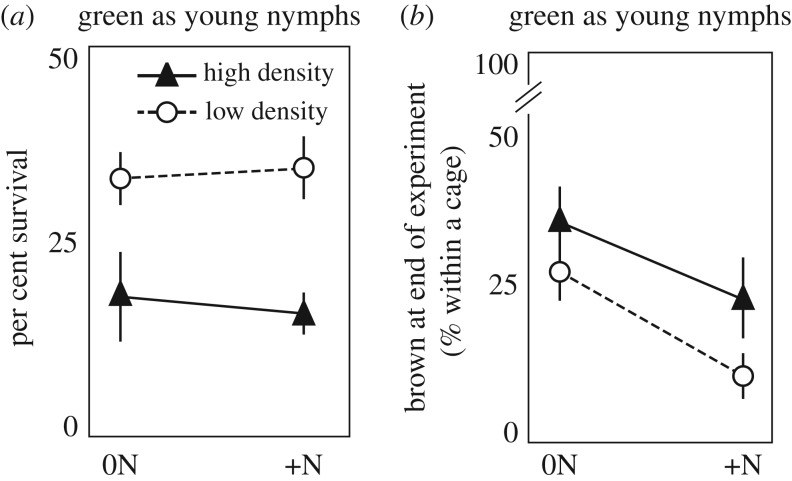
Per cent survival and colour change in green locusts reared in field cages. N fertilization had no effect on survival of green morphs (*a*). High density and low-N plants increased the per cent of brown morphs in cages where all locusts started as green morphs (*b*).

In cages where all individuals started as green juveniles (figure 3), there was no significant interaction between fertilization and density on survival (two-factor ANOVA *F*_1,38_ = 0.21, *p* = 0.65) and no main effect of N-fertilization (ANOVA *F*_1,38_ = 0.02, *p* = 0.90). However, high density decreased per cent survival of green juveniles (ANOVA *F*_1,38_ = 14.15, *p* = 0.001). Nitrogen fertilization promoted maintenance of green morphs (ANOVA *F*_1,38_ = 7.40, *p* = 0.01) while high density tended to promote switching to brown morphs (ANOVA *F*_1,38_ = 4.02, *p* = 0.05). However, there was no significant interaction of fertilization and density (two-factor ANOVA *F*_1,38_ = 0.21, *p* = 0.65) on the per cent of brown morphs per cage.

### 3.3. Effects of varying macronutrient ratios in artificial diets on survival and flight

**Figure 4.**
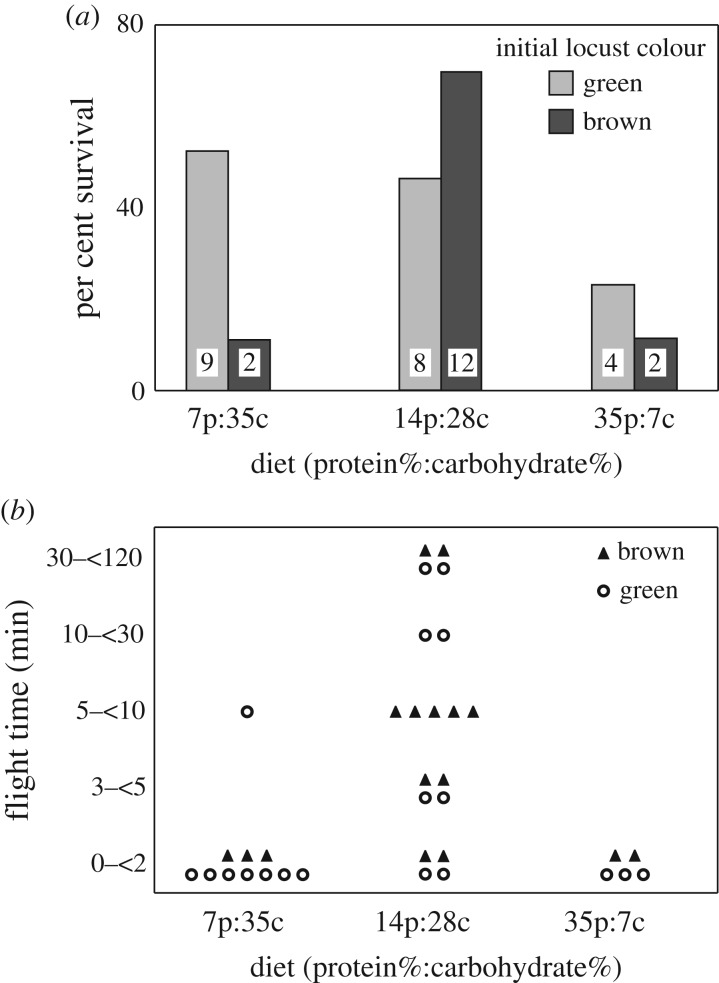
Per cent survival (*a*) and flight duration (*b*) in locusts fed synthetic diets with different ratios of protein and carbohydrate. Initial locust colour had a significant effect on survival across different diets (*a*), but there was no significant effect of initial colour on flight (*b*). Only locusts that were fed the 14p:28c diet, which was associated with high survival, showed appreciable tendency and ability to fly for extended periods. In panel (*b*), solid triangles represent brown locusts and open circles represent green locusts. Each symbol is one locust. Locusts were binned into one of five different flight durations ranging from 0 to 120 min of flight.

Locust phenotype influenced how diet affected survival, with the migratory brown morph most sensitive to diet quality. Brown locusts had high per cent survival when reared on the 14% protein:28% carbohydrate diet and low survival on both low- and high-protein diets (*χ*^2^ = 18.21, *p* < 0.001, d.f. = 2; figure 4*a*). By contrast, there was no significant difference in survival for the green locusts across different diets (*χ*^2^ = 3.4, *p* = 0.18, d.f. = 2), although green locusts on the highest protein diet tended to have the lowest per cent survival.

Locusts fed the 14 protein : 28 carbohydrate ratio diet were most likely to fly for longer than 5 min (*χ*^2^ = 10.4, *p* = 0.005, d.f. = 2; figure 4*b*). With the exception of one locust from the 7p:35c treatment, all locusts from the 7p:35c and 35p:7c treatments flew for less than 2 min. We selected the 5 min cut-off to be conservative, only counting locusts that flew longer than 5 min as having sustained flight activity. There was no effect of initial colour (green or brown) on flight time (*χ*^2^ = 1.18, *p* = 0.28, d.f. = 1). However, we only tested locusts 4–6 days post moult. It is possible that running this test on older locusts may reveal significant differences in flight capacity between green and brown morphs.

### 3.4. Effect of sex on locust response to different diets

For the laboratory-based studies, we were unable to run adequate replicates to include both sexes due to space constraints in the field station in rural Inner Mongolia. Therefore, these tests only include females. For the field-based studies using field cages, we used equal numbers of males and females. The low survival on N-fertilized field plots using both sexes corroborates the pattern we found in females reared in the laboratory. However, future studies are needed to determine whether males exhibit similar changes in migratory characteristics in response to different diets, as compared to females.

## 4. Discussion

In contrast to the hypothesis that performance-degrading food should enhance development of migratory characteristics [7,8], our results support the more nuanced hypothesis that high-quality diets promote development of migratory characteristics when migration is physiologically challenging. We suggest that because locusts, like many birds, fly long distances, high-quality food is critical for flight success. Non-optimal diets may make it difficult for locusts to achieve the physiological condition necessary for successful migration and may preclude development of migratory characteristics even when faced with intraspecific competition under crowded conditions. Moreover, we found that migratory phenotypes were more susceptible to diet quality variation, which may result in the non-migratory phenotypes having greater fitness than migratory phenotypes when the nutritional quality of host plants is poor. Our findings support recent calls for a renewed study of migratory and dispersal reaction norms [5] to aid in developing an understanding of the regulation and evolution of migratory and dispersal behaviours [6].

### 4.1. High density and high-quality diets enhance migratory morphology and colour

Rearing *O. asiaticus* at high density on low-N (performance-enhancing) grass resulted in morphs that had the most enhanced migratory morphology (figures 1 and 2). For this species, outbreaks and migratory swarms are comprised predominantly of brown morphs. Previously, we showed that brown morphs have a greater relative investment in thorax and hind legs when compared with green morphs, in addition to higher metabolic rates and activity levels [29]. Similar changes in body shape have been found in other insects exhibiting migratory polyphenism. For example, long-winged morphs of the common water strider (*Gerris remigis*) had larger thoraxes but smaller abdomens than short-winged morphs [39]. Roff & Bradford [40] compared shape differences among long-winged and short-winged crickets (*Allonemobius socius*) and found that long-winged crickets had larger femurs, wider rear pronota and narrower front pronota than short-winged crickets [40]. In some Lepidoptera species enhanced flight performance is correlated with higher thorax to body ratios (*Bicyclus anynana* [41]) and wing size (*Pararge aegeria* [42]).

In many locust species, darker coloration by melanization is indicative of gregarious morphs that form migratory swarms (e.g. *Schistocerca gregaria* and *Locusta migratoria*; reviewed in [43]). By contrast, solitarious locust morphs, which avoid each other and do not form swarms, tend to be lighter in colour and more cryptic. Previously, we showed that *O. asiaticus* fits with this pattern because outbreaks are predominantly made up of darker, brown locusts [29]. However, it was unclear if colour was developmentally plastic as it is in *S. gregaria* and *L. migratoria*. In our field cage study, locusts that started as green were induced to change to brown morphs in treatments with high density and unfertilized grass (figure 3). Previous research found no effects of density on locust colour if the animals started as brown [29]. This suggests that *O. asiaticus* will more readily shift from a green/non-migratory morph into a brown/migratory morph than shift in the opposite direction. The propensity to more rapidly develop into a migratory phenotype as opposed to the other way around has been shown for the desert locust (*Schistocerca gregaria*) both within and across generations [44] (reviewed in [43]). Results from the laboratory study (figure 2) showed that body shape can change over the course of a single juvenile instar in brown morphs (while colour did not change), suggesting that body shape can begin shifting towards a non-migratory form prior to the colour shift from brown to green. However, our laboratory studies only looked at effects within single instars; likely effects will be stronger with longer developmental exposure to different diets and densities. Future studies are needed to determine effects of longer durations of developmental exposure, and to test whether there are critical developmental stages for diet to modulate the effect of density on these different locust phase change characteristics.

### 4.2. High-quality foods support flight activity

Our results show that an optimally balanced diet motivates flight for Mongolian locusts, *O. asiaticus* (figure 4). These results are consistent with studies of the ecophysiology of avian migration where migrating birds may be considered extreme athletes and high food quality is considered an important determinant of a migrant's vitality [18]. Prior to and during migration, birds may shift to a carbohydrate and/or lipid biased diet to increase lipid stores. For example, two warbler species (*Sylvia atricapilla* and *Sylvia borin*) select fruits to maximize lipid intake [14] using fruit colour as a cue to indicate lipid content [15]. Red knots (*Caladris canutus*) feed on rich horseshoe crab eggs in Delaware Bay prior to migrating to the Arctic [16] and over-harvesting of crabs can threaten the capacity of red knots to refuel [17]. Similarly, long wing crickets (*Gryllus firmus*) preferentially ate carbohydrate-biased diets and had higher lipid concentrations relative to short-wing morphs [45] while migratory locusts (*Locusta migratoria*) that flew on flight mills increased consumption of carbohydrates [46]. Migrating locusts are generally more active, have higher metabolic rates and increased lipid stores, the primary fuel used for long-duration (more than 30 min) flight [47–51]. Likewise, our behavioural data suggest that carbohydrate-biased diets are required for *O. asiaticus* to exhibit flight activity persisting for more than a few minutes (figure 4*b*). Future studies are needed to determine what flight fuels *O. asiaticus* uses over which durations and how diet might influence that process.

Unlike the situation in these confined laboratory diet experiments, in the field, behavioural selection of preferred diets will allow locusts some capacity for seeking their preferred diet. In Inner Mongolia where *O. asiaticus* is prevalent, low-N *Stipa grandis* is its preferred host plant [30,52] and is consistently the plant with the lowest N concentration (e.g. [53]), which translates to a low crude protein to non-structural carbohydrate ratio [54]. However, our field cage experiments demonstrate that despite a diversity of plants available, nitrogen fertilization created nutritional conditions that blunted formation of the migratory phenotype, suggesting that behavioural compensation to achieve the preferred diet was not possible. A requirement for low protein, high carbohydrate diets for sustained flight activity may explain why feeding *O. asiaticus* high N plants suppressed development of migratory phenotypes, even at high population density (figure 2) and why the brown, outbreak morphs had poor performance on high-protein diets (figures 1 and 4*a*) [30]. By contrast, the green, non-outbreak morph was less sensitive to suboptimal diets (figures 3 and 4*a*), which suggests these non-migratory phenotypes have greater fitness than migratory phenotypes when the nutritional quality of host plants is poor. In our artificial diet studies, we found that diets imbalanced in either direction—too much protein or too much carbohydrate—decreased flight propensity in locusts reared at high density (figure 4*b*). These results suggest that *O. asiaticus* may live on the edge of its nutritional niche (preferring the lowest protein, highest carbohydrate plants available) and that there is a nonlinear relationship between the migratory phenotype and dietary protein to carbohydrate ratio.

Locusts and other migratory pests continue to have devastating impacts on agriculture, food security and livelihoods globally [4,55]. Our results suggest a novel means to mitigate the impacts of migrating Mongolian locust swarms by leveraging the connections between agricultural practices, soil nitrogen, migrating locusts and livelihoods via management practices that retain more soil nitrogen and maintain grasses in a nitrogen-rich state [30,55]. Our findings could be relevant to other locusts that have similar physiological profiles and that exhibit similar patterns of outbreaks on degraded landscapes.

## Data accessibility

Data are available from the Dryad Digital Repository: http://dx.doi.org/10.5061/dryad.1s53q [56].
